# Copper Nanoparticles and Reduced Graphene Oxide as an Electrode Modifier for the Development of an Electrochemical Sensing Platform for Chloroquine Phosphate Determination

**DOI:** 10.3390/nano13091436

**Published:** 2023-04-22

**Authors:** Francisco Contini Barreto, Martin Kássio Leme da Silva, Ivana Cesarino

**Affiliations:** Department of Bioprocess and Biotechnology, School of Agriculture, São Paulo State University (UNESP), Botucatu 18610-034, SP, Brazil; francisco.c.barreto@unesp.br (F.C.B.); martin.leme@unesp.br (M.K.L.d.S.)

**Keywords:** copper nanoparticles, reduced graphene oxide, chloroquine phosphate, electrochemical sensor, antimalarial drug

## Abstract

This study describes the use of copper nanoparticles (CuNPs) and reduced graphene oxide (rGO) as an electrode modifier for the determination of chloroquine phosphate (CQP). The synthetized rGO-CuNPs composite was morphologically characterized using scanning electron microscopy and electrochemically characterized using cyclic voltammetry. The parameters were optimized and the developed electrochemical sensor was applied in the determination of CQP using square-wave voltammetry (SWV). The analytical range for the determination of CQP was 0.5 to 110 μmol L^−1^ (one of the highest linear ranges for CQP considering electrochemical sensors), with limits of detection and quantification of 0.23 and 0.78 μmol L^−1^, respectively. Finally, the glassy carbon (GC) electrode modified with rGO-CuNPs was used for quantification of CQP in tap water; a study was carried out with interferents using SWV and obtained great results. The use of rGO-CuNP material as an electrode modifier was thus shown to be a good alternative for the development of low-cost devices for CQP analysis.

## 1. Introduction

The COVID-19 pandemic, caused by a new strain of the coronavirus, first found in the city of Wuhan in early December 2019 in China [1], has caused millions of deaths around the world. During this period, several drugs were tested and/or developed in order to control these infections [2]. Chloroquine (C_18_H_26_ClN_3_) was the first drug to be used in the prevention and treatment of all types of malaria and has a low cost, which allowed it to be produced on a large scale in an attempt to combat the pandemic [3]; however, in addition to the lack of proof of its effectiveness for this purpose, it may increase the risk of heart attacks [4]. Zurita et al. [5] classified chloroquine as harmful to aquatic organisms and environments and suggested that it may have long-term adverse effects [5,6]. Humans are also susceptible to the aforementioned problems, and the risks of exposure are related to the accidental consumption of water in leisure activities, the consumption of aquatic animals that grew up in contaminated regions, and the fact that studies demonstrate that these contaminants are not removed by traditional methods of water treatment and are distributed for later consumption [7,8]. According to Tarazona et al. [9], about 60% of all chloroquine consumed is eliminated in feces and urine.

Certain analytical methods have been proposed for the analysis of chloroquine and its derivatives, such as ultraviolet spectrophotometry [10,11,12,13], immunoassay [14], chemiluminescence [15], nuclear magnetic resonance [16], spectrofluorimetry [17], and capillary electrophoresis [18], and most studies’ methodologies are based on chromatography [19,20,21,22,23,24,25,26,27,28], including high-performance liquid chromatography (HPLC) [22,23,24,25,26,27] and gas chromatography [28]. However, some of these techniques require solid-phase extraction, liquid–liquid extraction, high-performance solvents (chromatography grade), and highly trained personnel [20,29], which makes it difficult to perform these techniques in some cases. Due to certain advantages of electrochemical sensors, such as high sensitivity, low-cost equipment, fast analysis, miniaturization capacity, and the possibility of performing in situ analysis [30,31,32], they have gained prominence in the development of methodologies and analytical protocols for the detection of these compounds.

The use of carbon-based materials (graphene, for example) has increased significantly since the 2000s [30,33,34]. Characteristics of extensive mechanical variety and strength, high thermal conductivity, high electronic transport, large surface area, high electrocatalytic activity, chemical resistance, and optical quality are interesting properties of these materials that lead them to be widely re-used in electroanalytical applications, acting as a potential replacement for silicone and diamond carbon in electrodes [30,33,34,35,36]. Graphene has the ability to be functionalized and create multifunctional properties [36] and has been extensively modified with metallic nanoparticles and applied in the determination of the most diverse types of contaminants, such as sulfamethazine [37], trimethoprim [38], sulfamethoxazole [39], and also estriol [35], and tryptophan [40], which are endocrine disruptors.

Different metallic nanoparticles (MNPs), such as gold, silver, copper, antimony, and palladium, are widely used in electrochemical sensors as work electrode modifiers due to the synergistic characteristics they perform, increasing surface area, conductivity, and stability [30,33,35]. Some MNPs, for example, copper, are low cost, which makes their use quite attractive, and they have been successfully applied in the analysis of estriol [35], isotretinoin [34], and glyphosate [41].

Aiming at greater sustainability compared to most of the methods used for the determination of this antimalarial, and reducing toxicity and market value compared to the use of more noble materials, this work offers a new approach to the analysis of environmental contaminants. In this study, copper nanoparticles and reduced graphene oxide were used to modify glassy carbon for the determination of chloroquine phosphate in tap-water samples. The composite was synthetized, characterized, and applied in the proposed analysis.

## 2. Materials and Methods

### 2.1. Instrumentation

A potentiostat (PGSTAT-128N Autolab Electrochemical System, Utrecht, The Netherlands) and NOVA 2.1 software (Metrohm, Utrecht, The Netherlands) were applied in the voltametric experiments (cyclic voltammetry (CV) and square-wave voltammetry (SWV)), as well as a conventional three-electrode glass electrochemical cell. The working electrodes used in the experiments were the glassy carbon (GC), reduced graphene oxide (rGO), and reduced graphene oxide with copper nanoparticles (rGO-CuNPs). The auxiliary electrode was a platinum plate and the reference electrode was Ag/AgCl/KCl (3.0 mol L^−1^).

To characterize the nanoparticles morphologically, we performed scanning electron microscopy (SEM) using equipment localized at IQ-UNESP Araraquara, Brazil. 

### 2.2. Solutions and Reagents

The solutions prepared for the experiments used ultrapure water (Millipore Milli-Q system with resistivity ≥ 18.2 MΩ cm^−1^). All reagents were of analytical grade and were not purified before being applied in the studies. Graphene oxide (GO), CuCl_2_ (anhydrous), estriol, methylparaben, and chloroquine phosphate (CQP) were obtained from Sigma-Aldrich.

### 2.3. Synthesis of Reduced Graphene Oxide (rGO)

The rGO-CuNP synthesis was performed according to a procedure described previously by our group [35]. Initially, a 10:4 proportion of a suspension of GO and sodium dodecyl sulfate was made in an ethanol medium. Next, the suspension was added to an ultrasonic bath for 30 min. Then, 16 mg of sodium borohydride (NaBH_4_) was added, and the mixture was placed in an ultrasonic bath for one hour. After this process, the solution was transferred to falcon tubes to be centrifuged for 10 min at 3000 rpm, and then the precipitate was washed with ethanol, and the material was obtained.

### 2.4. Synthesis of Reduced Graphene Oxide with Copper Nanoparticles (rGO-CuNPs)

For this synthesis, the same procedures as for the previous synthesis were performed up to the step in which the solution was placed into the ultrasonic bath for one hour. Then, we calculated the mass of graphene, and copper chloride (CuCl_2_) was added at 30% (m/m) in relation to its mass, and then it was diluted with ethanol. To incorporate the copper nanoparticles into the graphene sheets, the CuCl_2_ solution was added under constant stirring at one drop per second. After this step was complete, the solution was placed in a sonicator for 15 min and then centrifuged to separate the solid–liquid phase and cleaned with ethanol after the separation. Prior to the use of the composite to modify the electrodes, the material was placed in an ultrasonic homogenizer for 5 min to guarantee the homogeneity of the composite to be applied in the electrode modification.

### 2.5. Electrode Preparation

Prior to modification, the glassy carbon (GC) electrodes were polished with polystyrene with silicon carbide sandpaper with a 0.5 μm aqueous alumina until a mirrored surface was obtained. Then, they were placed in a beaker with ethanol to be sonicated for 5 min. After this process, they were sonicated in water for another 5 min. The GC electrodes were immobilized, and 10 μL of composite solution (rGO or rGO-CuNPs) was applied to their surfaces. Then, they were dried at 50 °C in an oven and taken out for the electrochemical procedures.

### 2.6. Sample Preparation and Analysis of Chloroquine Phosphate in Tap Water

Calculated values of the standard chloroquine phosphate solution were added to a 10 mL aliquot of tap water, giving a final concentration of 1.0 mmol L^−1^ of chloroquine phosphate. This sample was combined with the electrochemical cell with 20 mL of 0.1 mol L^−1^ phosphate buffer (PBS) pH 5.0. The chloroquine phosphate index was determined by adding three successive aliquots of the standard solution.

## 3. Results and Discussion 

### 3.1. Morphological and Electrochemical Characterization of the Materials

**Figure 1:**
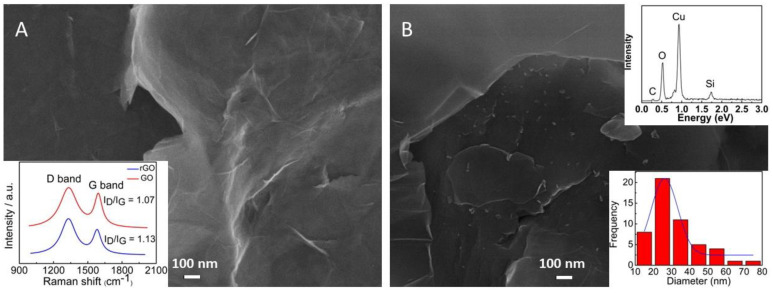
SEM images of (**A**) rGO material (inset: Raman spectra of GO and rGO material) and (**B**) rGO-CuNP nanocomposite (inset: EDS spectrum and histogram of the diameters of the nanoparticles).

rGO and rGO-CuNPs were morphologically characterized using SEM in order to analyze changes in the nanomaterials’ structures. Figure 1A shows rGO material, and it is possible to note a wavy, wrinkled, and twisted structure [34,35]. The graphene oxide is reduced to expose the sheets of carbon and create active sites, making it a more highly sensitive material in relation to the graphene oxide and making its characteristics more closely resemble those of graphene, which is a more expensive product. The inset of the figure shows the Raman spectra of GO and rGO, and the processes can be analyzed by the ratio between the G and D band. GO presented a ratio of 1.07, and the rGO presented a ratio of 1.13, and this change can be attributed to a higher size of sp^2^ domains and the chemical reduction [35]. Figure 1B shows the rGO-CuNP composite. It is possible to observe the copper nanoparticles on the rGO surfaces, whose diameters vary from 15 nm to 75 nm, providing evidence of the modification of the material. EDS was carried out to confirm the presence of Cu in the composite, and as can be seen, this element was incorporated into the material. 

**Figure 2:**
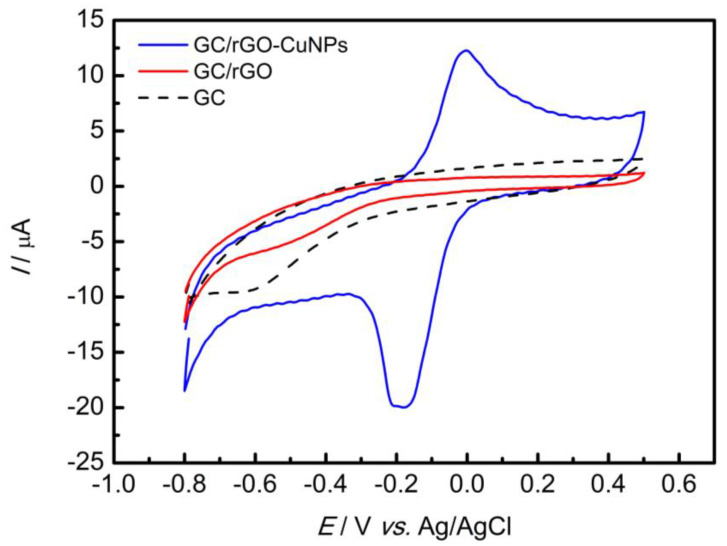
The GC, GC/rGO, and GC/rGO-CuNPs electrodes were electrochemically characterized by CV in PBS pH 7.0 with a scan rate of 50 mV s^−1^.

The electrochemical characterization of the GC/rGO-CuNPs was carried out by CV, in 0.1 mol L^−1^ PBS pH 7.0, with a scanning rate of 50 mV s^−1^, and the potential varying from −0.8 V to 0.5 V (Figure 2). In the cyclic voltammogram for the GC/rGO-CuNPs electrode (blue line), an oxidation–reduction process with well-defined peaks can be observed, which refers to oxidation (Cu^0^ to Cu^2+^) and reduction (Cu^2+^ to Cu^0^) of the copper, confirming the modification and incorporation of the nanoparticles into the material. The observed peaks are in agreement with previously published studies [35]. No electrochemical process was observed for the GC (dotted line) or GC/rGO (red line). 

### 3.2. Evaluation of the Electrochemical Behavior for the Different Working Electrodes

**Figure 3:**
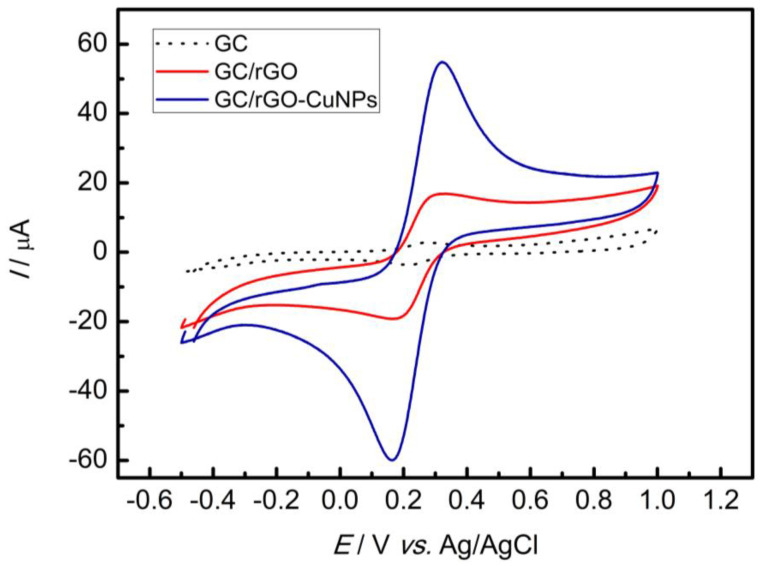
CV in the presence of 5.0 × 10^−3^ mol L^−1^ ferricyanide/ferrocyanide redox probe and 0.1 mol L^−1^ sulfuric acid solution with scan rate of 50 mV s^−1^ for the different types of electrodes.

In order to show the GC/rGO-CuNP electrode synergic effect compared to the GC and GC/rGO electrodes and to evaluate the composite efficiency regarding its conductivity, CV with a scan rate of 50 mV s^−1^, in the presence of 5.0 × 10^−3^ mol L^−1^ ferrocyanide/ferrocyanide redox probe and 0.1 mol L^−1^ sulfuric acid solution, was performed. As can be observed in Figure 3, the GC/rGO-CuNP electrode showed a higher peak compared to GC (17.45× and 16.09× higher in the anodic and cathodic peak, respectively) and GC-rGO electrodes (2.25× and 3.46× higher in the anodic and cathodic peak, respectively).

### 3.3. Electrochemical Behavior of the GC/rGO-CuNPs Sensor during the Chloroquine Phosphate Oxidation Process

**Figure 4:**
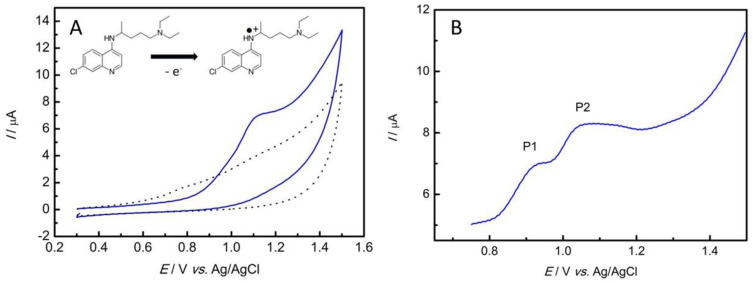
(**A**) CV recorded in 0.1 mol L^−1^ PBS (pH 7.0) with a scan rate of 50 mV s^−1^ in the presence of 100 μmol L^−1^ of CQP (blue line) and in the absence of the pharmaceutical (dark line). (**B**) SWV recorded in 0.1 mol L^−1^ PBS (pH 7.0) with a scan rate of 125 mV s^−1^, an amplitude of 20 mV, and a potential range of +0.75 to +1.5 V in the presence of 10 μmol L^−1^ of CQP.

The electrochemical oxidation of chloroquine phosphate on the GC/rGO-CuNP electrode was carried out in a 0.1 mol L^−1^ PBS pH 7.0 solution by CV with a scan rate of 50 mV s^−1^. As can be observed in Figure 4A, there was no electrochemical process in the absence of the antimalarial. However, with the addition of 100 μmol L^−1^ of CQP, an anodic peak at +1.1 V vs. Ag/AgCl/KCl can be observed. CQP can show one or two irreversible peaks [42], related to the oxidation of the N-heterocyclic nitrogen of the aminoquinoline portion and the nitrogen of the alkylaminoside group of the chloroquine molecule chain [42,43]. These peaks were better observed in the SWV (Figure 4B). For consistency and a better analytical response related to a higher anodic peak, the analyses were carried out with the second peak.

### 3.4. Optimized Parameters

Some parameters of the electrochemical study were optimized according to Table 1. In order to optimize the oxidation process of CQP, a pre-treatment experiment was carried out, varying the potential applied in a range of −1.2 to 0.8 V, and maintained for 30 s before the start of the SWV, with a scan rate of 125 mV s^−1^, an amplitude of 20 mV, and a potential range of +0.7 to +1.4 V. The potential that caused the best response in the current was −1.2 V.

It is known that different concentrations of composite on the electrode surfaces interfere with the quality of the sensor in the analysis of the molecules, and a large amount of the modifier material can block binding sites [35]. Therefore, the concentrations of the rGO-CuNPs were evaluated using SWV, with a PBS of 0.1 mol L^−1^ pH 7.0, a scan rate of 125 mV s^−1^, an amplitude of 20 mV, and a potential range from +0.7 to +1.5 V. As can be observed in Table 1, the best concentration used on the electrode surface was 0.04 mg/mL of rGO-CuNPs.

Finally, to analyze the oxidation process of CQP on the GC/rGO-CuNP electrode, the dependence of the electrochemical oxidation of chloroquine phosphate at different pHs was evaluated using SWV with PBS with pH varying from 5.0 to 9.0, an amplitude of 20 mV, a scan rate of 125 mV s^−1^, and potential range from +0.7 to +1.5 V. As can be seen in Table 1, the best pH to study CQP was 5.0, with the highest peak obtained in the analysis. Therefore, this obtained value was maintained in the subsequent analyses.

### 3.5. Analytical Characteristics

**Figure 5:**
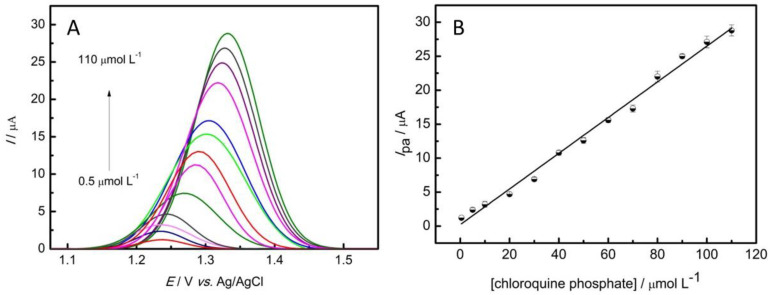
(**A**) SWV in 0.1 mol L^−1^ PBS pH 5.0 in the presence of varied amounts of chloroquine phosphate. (**B**) Linear relationship of anodic peak current as a function of chloroquine phosphate concentration.

An analytical curve was obtained in order to verify the linearity intervals, the limit of detection, and limit of quantification. Therefore, an SWV was used, with an amplitude of 20 mV, a scan rate of 125 mV s^−1^, a potential range from +0.7 to +1.5 V, and the optimized conditions described in Table 1. Then, the anodic peak was plotted with the respective CQP concentration. As shown in Figure 5, the technique presented a linear range between concentrations of 0.5 and 110 μmol L^−1^ of CQP with the following equation:

*I*_pa_ (μA) = 0.21 (μA) + 0.26 (μA/μmol L^−1^) × C_chloroquine phosphate_ (μmol L^−1^) (1)

The equation presented a coefficient of determination R^2^ = 0.992 (for *n* = 13 concentrations) with limits of detection and quantification of 0.23 μmol L^−1^ and 0.78 μmol L^−1^, respectively. The calculation was made using a 3σ/slope ratio and 10σ/slope ratio for the detection and quantification limits, where σ is the standard deviation of the mean value for 10 voltammograms of the blank. To perform a reproducibility test, the electrochemical measurements with chloroquine phosphate were performed in triplicate with three different modified electrodes, obtaining a value of 3.4%. 

The linear range obtained, when compared with the literature, has one of the largest differences between the highest and lowest value [2,42,43,44,45,46], which is useful for the analysis of the most diverse concentrations. The highest limit of 110 μmol L^−1^ is higher than many of the reported ranges as well [2,42,43,44,45,46,47]. Table 2 presents some works that used an electrochemical approach to analyze antimalarial drugs, with their method, linear range, LOD, and the analyte studied. This work presented one of the lowest LODs reported in relation to electrochemical methods. Compared to works that used Au in the electrodes, this work presented the advantage of the copper being less toxic and cheaper, and this material showed a linear range capable of analyzing greater concentrations. In relation to SnO_2_/graphite, the graphene material offers better chemical resistance and other qualities related to its characteristics [36], in addition to a higher range of linearity that allows the analysis of a greater range of concentrations. It is interesting to note that 2D carbon-based materials (such as graphene) tend to outperform other materials [30,33,34]. Therefore, the proposed electrode modification proved to be versatile and a good alternative for the determination of antimalarial drugs.

### 3.6. Determination of Chloroquine Phosphate in Tap Water

**Figure 6:**
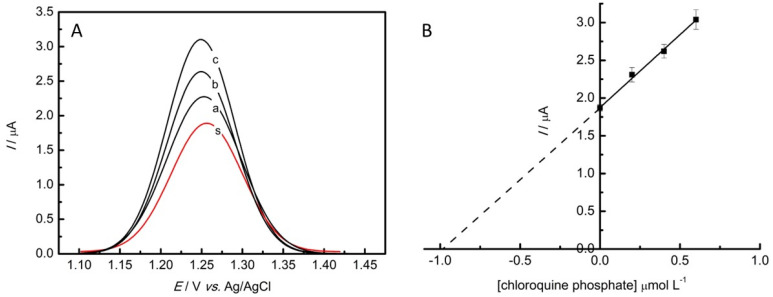
(**A**) SWV responses obtained for the determination of CQP in the tap water sample as follows: (s) sample; (a), (b), (c) represent the three successive additions of 0.2 mol L^−1^ of standard CQP. (**B**) Linear dependence of the anodic peak current with the CQP concentration of the standard addition method.

The GC/rGO-CuNP electrode was used for the quantification of chloroquine phosphate in the tap water sample. The determinations were performed in triplicate using the standard addition method, without any treatment procedure. Therefore, three additions of a known concentration of CQP (0.2, 0.4, 0.6 μmol L^−1^) were applied to 1 μmol L^−1^ of the sample in the electrochemical cell. The corresponding SWV voltammograms obtained for the analysis are presented in Figure 6. The results obtained for three determinations are listed in Table 3, and they had a mean of 1.030 ± 0.037 μmol L^−1^, with recoveries between 97.9% and 106.2%. The results indicate that rGO-CuNPs as an electrode modifier can be a good alternative for the determination of CQP in tap water in terms of its efficiency, stability, price, and toxicity. 

### 3.7. Determination of Chloroquine Phosphate in the Presence of Other Analytes

The influence of some interferents on the anodic peak of the developed sensor in the presence of phosphate chloroquine was investigated under the optimized conditions of the sensor. Estriol is a hormone that is found in aquatic matrices [35]; methylparaben [29] is commonly used in foods, pharmaceuticals, and personal care products and has been found in water matrices. Therefore, these analytes were used as interferents for the study of the recuperation of the signal of CQP. Firstly, a blank analysis was performed, followed by the visualization of the anodic peak of 1 μmol L^−1^ of CQP. Then, concentrations of 0.5, 1.0, and 2.0 μmol L^−1^ of the interferents were added to the electrochemical cell, and the anodic peak was measured. The results can been seen in Table 4. As can be observed, there were interferences in the signal of CQP in the presence of the interferents; however, more than 90% of the signal of the antimalarial could be detected by the developed electrochemical sensor, indicating that it could be applied in the water analysis contaminated with other molecules than CQP with great results.

## 4. Conclusions

Copper nanoparticles and reduced graphene oxide were used to modify a glassy carbon electrode for the determination of chloroquine phosphate. The rGO-CuNPs were characterized using scanning electron microscopy and electrochemically using cyclic voltammetry, which demonstrated the incorporation of the nanoparticles into the rGO. 

The parameters were optimized to provide a better response to the modified electrode and to improve the sensitivity in the analysis of the analyte. The LOD and LOQ obtained were 0.23 μmol L^−1^ and 0.78 μmol L^−1^, respectively, with a linear range from 0.5 μmol L^−1^ to 110 μmol L^−1^. 

Finally, the GC/rGO-CuNP electrode was applied in the determination of chloroquine phosphate in tap water, and a study with interferents was carried out. Excellent results were obtained, which indicates potential future applications for monitoring the indexes of this antimalarial drug in contaminated environments. 

## Figures and Tables

**Table 1:** Parameters optimized for the voltammetry analysis with the modified electrode based on nanoparticles.

Parameters	Optimization Range	Optimized Values
Pre-concentration (V)	−1.2–−0.8	−1.2
rGO-CuNP concentration (mg/mL)	0.01−0.10	0.04
pH	5−9	5

**Table 2:** Comparison of different electrodes reported in the literature for the determination of antimalarial drugs.

Electrodes	Method	Linear Range (μmol L^−1^)	LOD (μmol L^−1^)	Analyte	Ref.
ePADs	DPV	5−75	4.0	Chloroquine	[43]
GrRAC	DPV	5−65	1.05	Hydroxychloroquine	[2]
Glassy carbon	DPV	35−100	0.336	Hydroxychloroquine	[44]
β-CD-AuNP	DPV	0.01−0.05	0.00085	Hydroxychloroquine	[45]
SnO_2_/graphite	SWV	0.1–23.3	0.01	Chloroquine phosphate	[42]
rGO-CuNPs	SWV	0.5−110	0.23	Chloroquine phosphate	This work

**Table 3:** Results of the determination of CQP ^a^ in natural water samples using the proposed SWV method.

Repetition	Phosphate Chloroquine (μmol L^−1^) ^a^	Relative Errors (%) ^b^
1	1.062	6.2
2	1.050	5
3	0.979	−2.1
Mean ± SD	1.030 ± 0.037	

^a^ Added value for CQP: 1 μmol L^−1^. ^b^ SWV vs. added (SWV-added/added) × 100%.

**Table 4:** Effect of estriol and methylparaben on the anodic peak of 1 μmol L^−1^ of chloroquine phosphate by SWV in the developed sensor in optimized conditions.

Interferent	Concentration (μmol L^−1^)	% Chloroquine Phosphate Signal
Estriol	0.5	92.6
	1.0	92.4
	1.5	91.2
Methylparaben	0.5	96.4
	1.0	91.2
	1.5	90.3

## Data Availability

Data are available from the authors on reasonable request.
